# The Multifactorial Progression from the Islet Autoimmunity to Type 1 Diabetes in Children

**DOI:** 10.3390/ijms22147493

**Published:** 2021-07-13

**Authors:** Witold Bauer, Attila Gyenesei, Adam Krętowski

**Affiliations:** 1 Clinical Research Centre, Medical University of Białystok, Marii Skłodowskiej-Curie 24a, 15-276 Białystok, Poland; gyenesei.attila@pte.hu (A.G.); adamkretowski@wp.pl (A.K.); 2 Szentágothai Research Centre, University of Pécs, Ifjúság útja 20, 7624 Pécs, Hungary; 3 Department of Endocrinology, Diabetology and Internal Medicine, Medical University of Białystok, Marii Skłodowskiej-Curie 24a, 15-276 Białystok, Poland

**Keywords:** type 1 diabetes, T1D prediction, islet autoantibodies, HLA, gut microbiome

## Abstract

Type 1 Diabetes (T1D) results from autoimmune destruction of insulin producing pancreatic ß-cells. This disease, with a peak incidence in childhood, causes the lifelong need for insulin injections and necessitates careful monitoring of blood glucose levels. However, despite the current insulin therapies, it still shortens life expectancy due to complications affecting multiple organs. Recently, the incidence of T1D in childhood has increased by 3–5% per year in most developed Western countries. The heterogeneity of the disease process is supported by the findings of follow-up studies started early in infancy. The development of T1D is usually preceded by the appearance of autoantibodies targeted against antigens expressed in the pancreatic islets. The risk of T1D increases significantly with an increasing number of positive autoantibodies. The order of autoantibody appearance affects the disease risk. Genetic susceptibility, mainly defined by the human leukocyte antigen (HLA) class II gene region and environmental factors, is important in the development of islet autoimmunity and T1D. Environmental factors, mainly those linked to the changes in the gut microbiome as well as several pathogens, especially viruses, and diet are key modulators of T1D. The aim of this paper is to expand the understanding of the aetiology and pathogenesis of T1D in childhood by detailed description and comparison of factors affecting the progression from the islet autoimmunity to T1D in children.

## 1. Introduction

Type 1 diabetes (T1D) is a chronic endocrine disease that results from autoimmune destruction of insulin-producing β-cells in the pancreas after the asymptomatic period of various duration [1,2,3].

The development of T1D is a heterogeneous process, usually proceeded by the appearance of islet-specific autoantibodies against β-cells structures. Among the autoantibodies which are construed as a sign of ongoing β-cells destruction, islet cell cytoplasmic autoantibodies (ICA), and biochemical autoantibodies targeted to insulin (IAA), islet antigen-2 protein (IA-2A), glutamic acid decarboxylase (GADA) and zinc transporter 8 (ZnT8A) are the best characterised [4]. The two most common autoantibodies present at seroconversion in childhood are IAA and GADA, whereas IA-2A and ZnT8A autoantibodies appear as the first ones in a relatively small proportion. However, they are all common at the diagnosis of the disease [5,6]. Later in the disease, disturbances in glucose metabolism become more common as β-cell destruction proceeds.

The age of seroconversion differs between various autoantibodies initialising the autoimmunity, reaching its peak before the age of two for IAA, whereas GADA peaks at the age of four to five years and continues to appear at a relatively high level throughout childhood [7,8,9]. The risk of T1D increases with an increasing number of positive autoantibodies [10,11,12,13]. The observed risk of T1D is time-constant for high IA-2A levels but decrease over time for IAA and GADA [14]. Detailed analysis of this complex relationship, including also ZnT8 autoantibody, is still lacking. A small percentage of genetically susceptible children with islet autoantibodies do not progress to clinical T1D [10]. Other risk factors associated with the rapidity of disease development are genetic susceptibilities, defined by the T1D-associated HLA genotypes and non-HLA associated genes [15,16], age of the appearance of autoantibodies [5], sex [17,18] and probably still unknown environmental factors [9,19]. The varying length of the asymptotic phase suggests that environmental elements change the pace of disease progression in addition to genetic factors. The progression from seroconversion to the onset of clinical T1D and progression of islet autoimmunity is also known to be associated with the higher levels of especially IAA [20] and IA-2A [20,21] but also GADA [22,23].

The onset of clinical T1D can occur at any age, but the incidence is highest in childhood [24,25]. In 2019, 463 million people had diabetes, and over one million children and adolescents under the age of twenty had been estimated to have T1D. Type 2 diabetes (T2D) is the most common and accounts for the vast majority (over 90%) of diabetes worldwide. The appearance of clinical T1D is associated with a 40–70% decrease of β-cells mass, although these numbers differ in children and adult individuals with early stage and long-established diabetes [26]. The global incidence of T1D has been increasing continuously since the middle of the last century, although levelling off in incidence in populations with the highest increase has been recently described [27,28]. A decrease in the incidence of T1D in children in the Finnish population was reported recently both in boys and girls in the youngest age group [29]. The ability to predict at an early stage the risk of autoimmunity and T1D progression rates is crucial for selecting appropriately matched case–control groups for trials of preventive treatments. Early detection of autoimmunity is essential in studies on individual risk factors associated with the development of T1D. T1D causes an overwhelming burden on affected children and their families and puts a strain on the health system and society. Children affected with T1D have increased mortality as well as increased risk of cardiovascular comorbidity [30,31]. However, an early diagnosis leads to decreased complications at disease onset [32,33].

This review describes the genetic, immunological and environmental risk factors affecting the progression of islet autoimmunity and progression to clinical T1D in children. The articles presented in this review are limited to the most recent findings, coming from the longitudinal follow-up studies that track general populations, families of patients with T1D and young children at risk of T1D. 

## 2. Type 1 Diabetes in Children

### 2.1. Aetiology of Diabetes Mellitus

Diabetes mellitus includes a diverse group of metabolic diseases with the common feature of hyperglycaemia, which can be caused by insufficient insulin secretion and impaired insulin action on target tissues. The typical symptoms of hyperglycaemia include raised urination levels, thirst, weight loss and body fatigue. These symptoms may occur suddenly. Untreated diabetes can result in acute life-threatening complications, like diabetic ketoacidosis and secondary complications in advanced stages of the disease [34]. Diabetes patients have an increased risk of cardiovascular diseases and increased death rates [30,31]. Prior to the discovery of insulin therapy, a patient’s life expectancy with T1D was poor, and the disease was generally thought to be fatal. The etiologic classification of diabetes mellitus is complex. Most cases may be categorised into one of two major categories, type 1, or type 2 diabetes (T2D) [34], although more types of diabetes exist, including some caused by the genetic defects of both β-cell function and insulin action. T1D, which usually appears in childhood, is caused by autoimmune β-cell destruction, usually leading to absolute insulin deficiency. Immune dysregulation is caused by a combination of underlying genetic and environmental factors that enables autoreactive CD4+ and CD8+ T cells to destroy the pancreatic β-cells [35,36,37]. T2D is caused by a progressive loss of adequate β-cell insulin secretion frequently on the background of insulin resistance. The primary etiologic classification was suggested for the first time already in 1951, in a study in which the discrimination between insulin-dependent and non-insulin-dependent diabetes mellitus was stated for the first time [38]. 

### 2.2. Criteria for Diagnosis of T1D

Typical symptoms of T1D include excessive urination and thirst, which appear along with an unexplained weight loss. American Diabetes Association (ADA) and International Society for Pediatric and Adolescent Diabetes (ISPAD) [34,39] define clear recommendations for diabetes diagnosis. Unless there is an evident clinical diagnosis (children with classic symptoms of hyperglycaemia and a random plasma glucose ≥ 200 mg/dL (11.1 mmol/L)), diagnosis requires abnormal test results of fasting glucose ≥ 126 mg/dL (7.0 mmol/L), or plasma glucose ≥ 200 mg/dL (11.1 mmol/L)) at 2 hours during 75-g oral glucose tolerance test (OGTT). Traditionally, T2D was associated with the occurrence only in adults, while T1D was associated with the rapid development only in children. However, it has been reported that both diseases can occur in all age groups. T2D occurrence is associated with increased age (age ≥ 45 years old), excess body weight (BMI ≥ 25 kg/m^2^) and increased body fat percentage, although the exact threshold values differ in different race populations. Although autoimmune destruction of β-cells does not occur in T2D, at the time of diagnosis, some individuals cannot be clearly classified as having T1D or T2D. Although accurate classification is not straightforward at onset, the diagnosis of T1D becomes more apparent over time in patients with β-cell deficiency and in patients positive for the islet cell autoantibodies. Three distinct stages of T1D can be identified, with stage one defined by the presence of two or more of these autoimmune markers [34]. The persistent presence of two or more islet autoantibodies is a predictor of clinical hyperglycaemia and diabetes, according to the studies of the first-degree relatives of patients with T1D.

In T1D children, excessive urination and thirst are triggered by glucose levels exceeding the kidney glucose threshold. Due to β-cell destruction, insulin-sensitive target tissues, such as muscle and adipose tissues, do not receive enough glucose without insulin. In the absence of glucose, energy is produced from free fatty acids and amino acids, which causes weight loss and ketone accumulation. Other symptoms of diabetic ketoacidosis include feeling ill, tired and sleepy; the fruity smell of breath; increased heavy breathing, and confusion. 

### 2.3. Diabetes Prevalence 

In 2019, 463 million people had diabetes, and over one million children and adolescents under the age of twenty had been estimated to have T1D. According to the International Diabetes Federation, Europe has the highest number of children and adolescents before the age of twenty affected with T1D (296,500). Globally, the United States of America (175,900), India (171,300) and Brazil (95,800) have the highest numbers of children and adolescents with T1D. The incidence rates of T1D are highest in Europe, with over 31,000 new cases each year. In the group of children and adolescents before the age of fourteen, the top countries with the highest annual T1D incidence rates (per 100,000 population) include Finland, Sweden, Norway and United Kingdom in Europe, as well as Kuwait and Saudi Arabia [40]. 

Before the middle of the last century, the prevalence of T1D was low. The global incidence of T1D has been increasing continuously since the 1950s. However, in populations with the highest increase, the incidence has reached the plateau, and the gradual progress in incidence has been reported to slow down [27,28]. Currently, the disease incidence rates continue to rise at the rate of over 3% per year [28,40,41]. According to the estimates, the global number of new cases of T1D in a group of children and adolescents under the age of twenty is 128,900 annually [40]. The increase in the incidence rate was similar among boys and girls in the young and older children, but in tweens and young adolescents, it was significantly higher among boys than girls [28]. The high incidence of T1D among children highlights the need for novel research on preventing and delaying the onset of the clinical disease. Since the increase in the incidence rates of T1D is highest in developed countries and lowest in less developed countries, environmental factors are thought to contribute to the disease pathogenesis in addition to the well-established genetic factors. 

### 2.4. Prediction of T1D

The studies on risk factors associated with the development of T1D, precise prediction of progression of autoimmunity as well as ability to forecast the rates of progression to T1D at an early stage using cost-effective screening methods are crucial, given the high economic and social burden associated with the disease [42].

The data currently known about the pathogenesis of T1D comes from longitudinal follow-up studies that track preselected populations at risk of the disease. The choice of the study groups is mostly based on the genetic risk, family history or location of patients, prioritising people from countries with the highest prevalence of T1D. Prediction of T1D is typically based upon screening of HLA risk genotypes and islet cell autoantibodies precise measurements. Although these two techniques are the most reliable and cost-effective, islet cell autoantibodies can only be detected at a certain level when the progression of autoimmunity has already started. This emphasises the need for novel biomarkers of β-cells dysfunction that could be detectable before the beginning of the autoimmune response. Some of the most extensive prospective studies that focus on populations at risk of T1D are currently run in Finland, Sweden, Germany and the USA. These included the general population studies, families of patients with T1D or young children at risk of T1D. The All Babies in Southwest Sweden (ABIS) study is the most extensive prospective study in Sweden, with over 16,000 subjects enrolled in a cohort [43]. Some of the largest studies that have followed patients with an increased genetic risk of T1D at different ages are The Finnish Type 1 Diabetes Prediction and Prevention (DIPP) study in Finland [44], The Environmental Determinants of the Diabetes in the Young (TEDDY), which is a joint effort of USA, Finland, Sweden and Germany [45], The Diabetes Prediction in Skane (DiPis) in Sweden [46], and The Diabetes Autoimmunity Study in the Young (DAISY) study in the USA [47]. The DIPP and TEDDY projects are both ongoing prospective studies. In addition to this, the relatives of patients at risk of T1D are being followed in several studies, including the German BABYDIAB-BABYDIETY study [48] and an ongoing international TrialNet Pathway to Prevention [49] programme. Precise identification of subpopulations at risk of the disease may allow for personalised prevention clinical trials.

The process of autoimmunity progression and T1D is very heterogeneous, with some of the risk factors still unknown (Table 1) [3]. The root causes of T1D are complex, and novel approaches, besides traditional screening methods, are required to predict the disease onset accurately. In order to reveal potential environmental factors associated with the development of T1D, in 1986–1989 Childhood Diabetes in Finland (DiMe) study was carried out in siblings of children with T1D [50]. DiMe revealed several risk factors associated with the pace of progression of autoimmunity and the development of T1D, such as early exposure to cow’s milk formula-feeding and rapid growth in infancy. Systematic patient monitoring also allowed for early detection of new incidences of T1D, particularly among children. 

## 3. Factors Affecting the Progression of Autoimmunity

### 3.1. Genetic Factors Associated with the HLA Region

Although over 60 individual genetic loci have been associated with T1D in several studies [148,149,150], the polymorphisms of the HLA region remain the most significant contributors to the genetic susceptibility to T1D [151]. The HLA loci of DNA is approximately 4 Mb long and contains over 200 identified genes. The HLA region genes that are involved in the autoimmune response and are known to be linked to the progression to T1D can be divided into two categories: three genes that encode class I α-chain, (A, B and C), and three gene pairs of class II α- and β-chains (DR, DQ and DP) antigens. HLA class II loci are mapped to the centromeric end of the short arm of chromosome 6, while highly polymorphic class I loci are located at its telomeric end. Genes encoded by the class I HLA DR-DQ gene pairs can form four different types of class II molecules. The products of HLA class I and II loci genes are structurally similar molecules located on the cell surface, which function is the presentation of the peptide antigens to T lymphocytes. HLA class I antigens are responsible for CD8+ T cells presentation, while HLA class II antigens take part in a presentation to CD4+ T cells, which help B cell and CD8+ T cell responses. [152]. 

Although the HLA class II loci of chromosome 6 are highly polymorphic, different alleles are in strong linkage disequilibrium (LD) and form distinctive haplotypes. The HLA class II antigens combinations can form both protective (OR 1) and high-risk haplotypes [54]. The genetic risk of T1D is determined mainly by class II HLA DR-DQ haplotypes inherited from parents [153]. Polymorphisms in HLA-DRB1, DQA1 and DQB1 genes are strongly linked to T1D [154]. The two significant haplotypes associated with the risk of T1D are DR3-DQ2 and DR4-DQ8 [51]. The heterozygous DR3-DQ2/ DR4-DQ8 genotype is associated with the highest risk of progression from islet cell autoimmunity to clinical T1D [52]. Due to the strong LD, some haplotypes are inherited more often than others and might be overrepresented in specific populations. The risk-associated DR4-DQ8 and DR3-DQ2 are the most common haplotypes in Finland, partially explaining this population’s high T1D incidence rate [53]. The HLA class II genotypes have been linked to the expansion of autoimmunity in children with both IAA- and GADA-initiated autoimmunity [5]. The frequencies of both susceptibility and protective DR-DQ haplotypes are changing in recent years. Due to the increasing contribution of environmental factors, especially in developed countries, protective and mild-risk HLA haplotypes appear more often in T1D children [155]. 

Three HLA class I antigens (HLA-A, -B and -C) are encoded as a single α-chain. Several loci outside the HLA class II region have been associated with the increased risk of T1D [55,156]. Due to the strong LD of class I alleles and HLA class II loci, the exact association of class I antigens and progression to T1D was not easy in the past [157,158]. Independently from class II HLA, class I -A and -B alleles have been shown to play a crucial role in the age of onset of T1D [55,56,57,58]. Similarly, to the previous findings, in the Finnish population, polymorphisms of the HLA class I alleles have been linked to the age of onset of T1D and disease progression pace. HLA-B alleles are associated with increased T1D risk [159], suggesting that the HLA class I genes could play an essential role in the modulation of the β-cell destruction [54,59,60].

### 3.2. Genetic Factors Outside the HLA Region

More than 40 loci outside the HLA region of chromosome 6 have been identified as modifiers of clinical T1D risk. The identification of novel non-HLA genetic risk factors was possible thanks to the large-scale genome-wide association studies (GWAS) performed in recent years [151,160,161,162,163,164,165,166,167].

The strongest genetic association between T1D and nucleotide polymorphisms has been reported in the promoter region of the gene coding for insulin (*INS*). The short repeated VNTR region within the *INS* gene is significantly associated with the disease progression [61,62]. Other loci associated with T1D were rs698 and rs3842753 SNPs within the *INS* gene [63,64,65]. The polymorphisms in the *INS* gene are associated with higher insulin expression in the pancreas, which may affect the tolerance to insulin by regulating the insulin mRNA and protein expression [168,169].

Apart from the polymorphisms in the *INS* gene and few other loci: *PTPN22*, *SLC30A8* [66], and *BACH2* gene [16], no other SNP increase the risk of T1D with the odds ratio (OR) over 1.5. These findings highlight the importance of the HLA region compared to other genetic factors in the development of T1D [170]. Protein tyrosine phosphatase, non-receptor type 22 (*PTPN22*) gene polymorphism (rs2476601), is significantly associated with the progression from islet autoimmunity to clinical T1D [67] and rs45450798 in *PTPN22* is affecting the β-cell destruction early after the initial seroconversion [16]. Substitutions in the *PTPN22* gene that cause amino acid changes affect the B cells and T cells. In addition, it alters the function of immune cell signalling and impairs the function of regulatory T cells (Treg), which are essential in the pathogenesis of T1D [171,172]. Although most individual polymorphisms do not significantly predict the progression of autoimmunity, a genetic risk score (GRS) has been successfully applied to predict disease progression. GRS calculated as a weighted sum of all individual SNPs-associated risks has been reported to predict progression from islet autoimmunity to T1D in children and T1D progression pace in numerous studies [69,70,71]. Altogether, the genetic factors outside the HLA region and HLA genes are responsible for over 80% of the heritability of T1D [69,150].

### 3.3. Islet Cell Autoantibodies

Islet cell autoantibodies (any autoantibodies targeting the pancreatic islets of Langerhans) are some of the earliest markers of β-cell dysfunction. However, islet autoantibodies do not have any pathological functions [72]. Among biochemical autoantibodies, those targeting insulin (IAA), islet antigen 2 protein (IA-2A), glutamic acid decarboxylase (GADA) and zinc transporter 8 (ZnT8A) are the best characterised [4]. The non-specific islet cell cytoplasmic autoantibodies (ICA) are historically the first discovered [173,174]. 

The two most common autoantibodies present at seroconversion in childhood are IAA and GADA, whereas IA-2 and ZnT8 autoantibodies usually do not appear as the first one [20,84,175]. However, they are all common at the diagnosis of the disease [5,6]. The data obtained from longitudinal studies that have tracked populations from birth to the early onsets of clinical T1D indicate a high incidence of islet autoantibodies’ appearance already during the first year of life [18,20,72,74]. In the DIPP study, IAA appeared most often as the first autoantibody at the age of two [20].

The age at seroconversion differs between various autoantibodies initialising autoimmunity. The peak is before the age of two years for IAA, whereas GADA peaks appears at the age of three to five years and continue to appear at a relatively high level throughout childhood [7,8,9]. Young age at autoantibody appearance increases the risk of T1D [12,18,20]. The risk of T1D increases significantly with an increasing number of positive autoantibodies [10,11,12,13,20,72]. Rapid multiple autoantibodies development is mainly linked to high disease risk [20,72,73].

The order of autoantibody appearance affects the disease risk [175]. In slow progressors to T1D, GADA is the most frequent islet autoantibody to appear as the first one [80,81]. In children positive for multiple autoantibodies, GADA-initiated autoimmunity has been associated with a reduced risk of progression to diabetes [82]. ZnT8A positivity at a young age has been associated with delayed progression to T1D [80]. However, children positive for IA-2A are at increased risk of the disease [82]. IA-2A autoantibodies are associated with a high risk of progression to clinical disease [21,84,85]. IAA frequently appears among the first autoantibodies or as the single autoantibody [18,84,176,177]. The association between young age at seroconversion for IAA and high risk of T1D is well-established [20,74,75]. Additionally, a strong reverse correlation between IAA levels and age at primary seroconversion has also been reported. IAA levels measured three months after seroconversion are decreasing significantly with increasing age at seroconversion, and in the case of GADA, the decrease in autoantibody level with time is less apparent. However, the age at seroconversion has not been reported to influence the levels of IA-2A as the first autoantibody [14,20,21,22,23,88]. 

The HLA-associated genetic risk affects both the number and levels of islet autoantibodies [178]. The HLA class II genotype has a very similar effect on the expansion of autoimmunity in children with IAA and GADA-initiated autoimmunity [5]. IAA autoantibodies measured at seroconversion are significantly associated with HLA class II DR4-DQ8 risk haplotype [7,18,76,77,78,79] and polymorphisms of insulin-coding and *PTPN22* genes [16,179,180,181,182]. Similarly, IA-2A levels are significantly associated with the high-risk DR4-DQ8 haplotype [86]. GADA levels measured after seroconversion are significantly associated with the genetic risk of HLA class II genotypes [53]. Moreover, GADA autoantibodies appear significantly more often at seroconversion in children with HLA-DR3-DQ2 haplotype [6]. ZnT8A is associated with the accumulation of insulin [83]. No significant associations between HLA class I and II genotypes and persistent ZnT8A development have been reported. However, polymorphisms in the ZnT8 protein-coding *SLC30A8* gene, which affects insulin production, have been reported to be associated with T1D [68]. 

The progression from islet autoimmunity to the onset of clinical T1D is associated with higher levels of IAA [12,20], IA-2A [7,20,21,87,88] and also GADA [13,23]. Although, in the TEDDY study, GADA levels did not increase the risk of T1D [87]. The observed risk of T1D is time-constant for high IA-2A levels but decrease over time for IAA and GADA [14]. In recent studies, the levels of biochemical autoantibodies have been reported to be significantly higher in children who later progressed to T1D compared to non-progressors [14,20,21,22,23,88]. The autoantibody levels rise for the first year after autoantibody appearance in children who progress to diabetes or multiple autoantibodies after initial seroconversion. One year after the initial seroconversion, autoantibody levels are levelling off or declining, especially in the case of IAA. In the TrialNet study, high GADA levels were significantly associated with the overall risk of developing multiple autoantibodies [22,89]. Other studies have also reported that the levels of islet autoantibodies can be used for accurate T1D prediction [21,74,90].

### 3.4. Autoreactive and Regulatory T Cells

Although islet cell autoantibodies are the primary markers of progression of the disease progression, autoantibodies alone do not affect the β-cell damage [100]. T1D is caused by autoreactive T cells that activate and kill the β-cells in the pancreas, resulting in insulin insufficiency and hyperglycaemia. According to the recent findings, the uncontrolled activation and expansion of autoreactive CD4+ and CD8+ T cells in T1D are caused by the defects in immunosuppressive regulatory T cells (Treg), which can suppress activated autoreactive cells. In heath, potentially pathogenic autoreactive T cells, with specificity for islet autoantigens, are held in check by multiple regulatory mechanisms, including Tregs. Tregs are a specialized subpopulation of T cells that maintain homeostasis and self-tolerance by suppressing immune response [91]. Since the precise differentiation between subsets of regulatory cells in humans is not trivial, transcription factor forkhead box P3 (FOXP3) Tregs term is often used to refer to both Tregs generated from a subset of CD4+ in the thymus, as well as Tregs generated from the naïve CD4+CD25- T cells in the periphery [92]. Both subsets of FOXP3-positive Tregs can inhibit and downregulate effector T cell proliferation and cytokine production and play a critical role in preventing autoimmunity [183,184]. Repairing the defects in Treg in order to restore the defects in immune system regulation is a potential strategy for disease treatment [185]. 

Autoreactive T cells are the primary mediators that are likely to contribute to the pathogenesis of T1D [186]. T-cell subsets might be useful as biomarkers of treatment efficacy in clinical trials [187]. T helper cells are increased in number before and at diagnosis of type 1 diabetes and might be helpful as biomarkers for disease prediction [188,189]. The most specific markers of Treg cells are FOXP3, CD4 and CD25. Alterations in CD4 T cells have been reported in patients with T1D. Similarly, the frequency of T helper cells has been reported to be increased in multiple autoantibody-positive children [190,191,192]. Dysregulation in Treg cells frequencies or functions may lead to the development of autoimmune diseases, including T1D [92,93]. Functional deficiencies of Treg in T1D are associated with T1D progression [94]. Changes in subsets of Treg might be related to more advanced stages of T1D progression [93]. Alterations in Treg profiles lead to the dysfunction of the immune regulatory mechanisms critical for protection from T1D-associated autoimmunity. FOXP3 is necessary for the proper function of Treg, and their dysfunction might lead to immunodysregulation polyendocrinopathy enteropathy X-linked syndrome, which is often characterized by autoimmune enteropathy and T1D [193]. Alterations in FOXP3 Treg profiles have been associated with T1D and might serve as the potential biomarkers of the disease progression [91,95,96].

### 3.5. Environmental Risk Factors

In T1D, various environmental factors can result in the progressive loss of β-cell function that manifests clinically as hyperglycaemia. T1D is an autoimmune disease caused by an interplay of genetic and environmental factors. Several genetic risk and protective factors, mainly associated with the HLA genotypes, have been identified using genome-wide association studies during the past decades. It was speculated that the genetic predispositions of an individual solely drive the progression of autoimmunity. However, genetic predisposition alone is not sufficient to explain the increase in the prevalence of T1D since the 1950s. Several hypotheses propose an explanation for the rise in the prevalence of T1D [194,195,196]. Additional environmental factors explain the increase in frequencies of class II HLA genotypes in the general population in recent years [197,198].

Having first-degree relatives affected by T1D increases the risk of the disease compared with the more often sporadic cases of T1D. Children with familiar disease have IAA autoantibodies appearing more frequently than children with sporadic T1D [100,101]. Interestingly, having an affected father compared to an affected mother is linked to more frequent severe diabetic ketoacidosis and weight loss in children affected by T1D. Paternal T1D is associated with more severe disease progression [102]. 

Environmental factors, mainly those associated with changes in the gut microbiome, are a key modulator of T1D, though its precise role in the progression of autoimmunity is unclear [111,112]. Living environment, microbial contact, and taxonomic and functional changes in the gut microbiome of diabetic patients have been of interest in recent years, especially the association between the gut microbiome and immune system and the pathogenesis of T1D. 

### 3.6. Gut Microflora

The gut microbiome can be affected by multiple factors while simultaneously maintaining its homeostasis. At childbirth, the gut microbiome of infants is largely dependent on the delivery method, and there are significant differences between the infants delivered through C-section and vaginally delivered. In infants born through C-section, the gut microflora is similar to the mother’s skin surface microflora. In contrast, in those vaginally delivered, it is closer to the mother’s vaginal microbiome [199]. Infants delivered through the C-section were also associated with reduced taxonomic and functional diversity of its gut microbiome [103].

During the first years after childbirth, a child’s immune system gains sufficient immunological memory by intense education through numerous microbial contacts and infections. It is the period when the gut microbiome depends mainly on diet, drugs, antibiotics usage and even emotional stress. Serious life-threatening events and childhood emotional stress is associated with a higher risk of T1D in children [97,98,99]. Some of the other known factors that affect the diversity of infants’ gut microbiome are breastfeeding time, presence of solid foods in the early diet and introduction of antibiotics [200]. In addition, breastfeeding while introducing solid foods has been reported to decrease the risk of T1D [104]. The composition of the child gut microflora stabilises by the age of three and afterwards behaves similarly to this in adults [201]. Maturation of the gut microbiome coincides with the development of an immune system and the appearance of the first autoantibodies associated with T1D [202].

Changes in the taxonomic composition of the gut microbiome precede the appearance of islet autoimmunity [203]. These taxonomic changes in the gut microbiome composition result in the decreased diversity of gut microbes in T1D. Children that are positive for at least one islet cell autoantibody and those who later during the follow-up progress to T1D have a higher *Bacteroidetes/Firmicutes* ratio and lower Shannon diversity index of the gut microbiome compared to the healthy individuals [113,114]. Similarly, decreased diversity of gut microflora was observed when autoantibody-positive children *before* and *after* the onset of clinical T1D were compared [115]. A higher abundance of *Bacteroides* is common in children positive for at least one islet cell autoantibody [116], and progressors to T1D [117,118]. Data coming from the longitude follow-up studies demonstrate that alterations in the gut microbiome, which can be independently affected by multiple factors, are associated with the early development of islet cell autoantibodies [119]. It is being speculated that chronic fluctuating changes in the taxonomic composition of gut microflora could lead to system dysregulation and trigger immune responses, which lead to the progression to autoimmunity. However, this hypothesis has not been confirmed.

### 3.7. Viral Infections

Several pathogens, especially viruses, may be involved in the progression of autoimmunity and T1D development. Some studies have shown that viral infections, mainly those by enteroviruses, could be involved in the pathogenesis of T1D. Because of the molecular mimicry of human islet cell autoantigens, Coxsackie B virus and enteroviruses, which could be found in the pancreatic islets of most patients with T1D, could speed up the disease progression through the activation of the immune system [124,125,126,127]. The enteroviruses may also cause an acute infection of the pancreatic β-cells, resulting in β-cell destruction and progression to clinical T1D [128].

Several epidemiological studies supported the role of viruses, other than enteroviruses, especially rotaviruses [129,130], in progression to clinical T1D. An increased risk of disease progression in children affected with rotaviruses stays in line with the recent findings in the USA and Australian populations. In these two countries, a decrease in the prevalence of T1D was observed shortly after rotavirus vaccinations recommendations were introduced [129,130]. The association between the decreased T1D risk and other vaccinations have been reported [204] especially in the context of measles vaccination. The risk of T1D is significantly lower in measles vaccinated children [131]. Similarly, the influenza vaccination was associated with lower T1D risk in the TEDDY study [132]. 

During the COVID-19 pandemic of 2020, several studies have investigated the potential association between the severe acute respiratory syndrome coronavirus 2 (SARS-CoV-2) infection and the prevalence of T1D in children and progression to diabetic ketoacidosis [205,206,207,208,209,210,211]. The evidence for the insulin-producing β-cell damage by the virus has been reported [133].

### 3.8. Dietary Factors 

Diet is another environmental factor that affects the progression from islet autoimmunity to clinical T1D. Early exposure to cow’s milk is associated with more rapid progression to T1D. One hypothesis explaining the role of cow’s milk in disease progression is albumin’s molecular mimicry to ICA, a surface protein of pancreatic β-cells [134]. High consumption of cow’s milk in childhood has been associated with an increased risk of progression from islet autoimmunity to T1D [135,136,137]. The effect of hydrolysed infant formula versus conventional formula on the risk of T1D was studied in the TRIGR Randomized Clinical Trial [212]. However, no effect of the hydrolysed infant formula consumption on the risk of T1D was found. 

An increase in the prevalence of T1D since the 1950s is often linked to Western civilisations’ diet and eating habits. Changes in children’s eating habits include early exposure to processed food, the introduction of gluten-containing food and cow’s milk, short breastfeeding and formula feeding. These dietary changes may partially explain a recent increase in diabetes prevalence in developed and developing countries compared to the rest of the world. Higher consumption of sugars and carbohydrates have been associated with more rapid progression of T1D in the DAISY follow-up study in 2015 [138].

The studies of potential dietary contributors to T1D were primarily focused on the infancy period. Still, it has been shown in several studies that maternal diet during pregnancy and the breastfeeding period may also influence the progression of islet cell autoimmunity. Short breastfeeding time is associated with an increased risk of progression of autoimmunity [105,106]. Early introduction of gluten-containing solid foods is associated with an increased risk of islet cell autoimmunity [107,108,109,110]. Interestingly, early exposure to gluten-free oats increases the risk of T1D in a similar fashion [213]. Children exposed to gluten-containing cereals, which were simultaneously breastfed, were less likely to progress to T1D, which emphasise breast milk’s role in early immune system development [104].

Birth weight and weight gain during the first year of life are associated with the risk of T1D [139]. Low birth weight has been associated with decreased risk of T1D, and an increased weight gain in infancy has been associated with an increased risk of T1D [140,141,142]. The prevalence of obesity has been linked to the increased risk of T1D in DiMe children [143]. In more recent studies, a significant association between body weight and selected HLA genotypes and progression of islet autoimmunity has been reported [144,145,146]. In children under the age of 10, overweight has been reported to be positively associated with an increased risk of progression from islet autoimmunity to type 1 diabetes and with the development of type 1 diabetes, but not with the development of autoantibodies [147]. 

Increased vitamin D consumption and higher serum 25-hydroxyvitamin D levels are considered protective factors against T1D [120,121]. The deficiency of vitamin D may increase the risk of the disease [122,123]. However, in the longitudinal follow-up DIPP study, no association has been found between cord blood 25- hydroxyvitamin D concentrations and islet autoantibodies development and progression to T1D [214,215]. No association of vitamin D intake and islet autoimmunity was also reported in the DAISY study [216].

## 4. Conclusions

The disease progression factors after the diagnosis are not fully understood, and the process has been proven highly heterogeneous. In addition to the well-established factors, including supplementary cow’s milk formula feeding, milk consumption, viral infections and alteration in the gut microflora, numerous environmental, social, and economic factors can affect the pathogenesis of this disease in children. The gene-environmental interactions exist, and the underlying causes of T1D are complex and multifactorial (Table 1). 

The increase in the prevalence of T1D in recent years emphasises the need for new, reliable, and cost-effective disease prediction methods. To date, the individuals at high T1D risk can already be identified reliably by HLA risk genotypes screening and islet autoantibodies profiling. These two screening methods are the most reliable strategies for the prediction of T1D. Screening for HLA high-risk genotypes is easily accessible in all cases. However, islet cell autoantibodies can only be measured at a certain stage of disease progression, at which the humoral autoimmunity has already been engaged. Thus, novel approaches besides traditional screening methods are required to predict the disease onset accurately, before the first signs of islet cell autoantibodies appear. Changes in the taxonomic composition of the gut microbiome, which are currently studied in children as potential biomarkers of T1D, precede the appearance of islet autoimmunity

Identifying factors leading to the destruction of β-cells offers potential means for intervention aimed at preventing T1D. It is already possible to manipulate the spontaneous appearance of islet autoantibodies by dietary modification early in life. Other attractive therapeutic targets for T1D treatment are Tregs, of which low number and impaired functionality are characteristic already at the disease onset. A transfer of ex vivo expanded Tregs back to the patient might be a potential approach in T1D treatment. This innovative approach is supported by the early evidence coming from clinical trials in kidney transplantation [217,218,219]. T1D causes an overwhelming burden on the families of affected children and puts a strain on the healthcare system. Children affected with T1D have increased mortality as well as increased risk of cardiovascular comorbidity, but thanks to an early diagnosis, complications at disease onset may be decreased.

## Figures and Tables

**Table 1:** Main categories of factors affecting the progression of islet cell autoimmunity and T1D development.

Risk and Protective Factors	Reported Effects and Associations	References
HLA class II DR3-DQ2 and DR4-DQ8 haplotypes	Increased risk of rapid progression; expansion of autoimmunity in children with both IAA- and GADA-initiated autoimmunity	[5,51,52,53]
HLA class I -A and -B alleles	Modified age of onset of T1D; increased risk of T1D; modulated the β-cell destruction	[54,55,56,57,58,59,60]
*INS*, *PTPN22*, *SLC30A8*, and *BACH2* gene SNPs; alterations in the VNTR region within the *INS* gene	Increased progression from islet autoimmunity to clinical T1D; β-cell destruction early after the initial seroconversion	[16,61,62,63,64,65,66,67,68]
High Genetic Risk Score	Prediction of T1D; increased progression from islet autoimmunity to clinical T1D; increased disease progression pace	[69,70,71]
Young age at autoantibody appearance	Increased risk of T1D	[12,18,20]
Positivity to multiple autoantibodies	Increased risk of T1D; association with the extremely high-risk of T1D	[10,11,12,13,20,72,73]
Seroconversion to IAA	Increased risk of T1D; association with young age at seroconversion and increased risk of T1D; reverse correlation between IAA levels and age at primary seroconversion; association with HLA class II DR4-DQ8 risk haplotypes	[7,18,20,74,75,76,77,78,79]
GADA present at seroconversion	Decreased risk of T1D in multiple autoantibody-positive children; association with the slow progression to T1D; association with protective HLA-DR3-DQ2 haplotype	[6,80,81,82]
Seroconversion to ZnT8A in young age	Delayed progression to T1D; no association with HLA class I and II haplotypes	[68,80,83]
Seroconversion to IA-2A	Increased risk of T1D; association with the high-risk DR4-DQ8 haplotype	[21,82,84,85,86]
High levels of IAA, IA-2A and GADA autoantibodies at seroconversion	Rapid progression from islet autoimmunity to the onset of clinical T1D; increased risk of developing multiple autoantibodies; accurate prediction of T1D	[7,12,13,14,20,21,22,23,74,87,88,89,90]
Dysregulation or functional deficiencies of FOXP3-positive Treg	Development of autoimmune diseases, including T1D; more severe disease progression	[91,92,93,94,95,96]
Serious life-threatening events and stress	Increased risk of T1D	[97,98,99]
First-degree relatives	Increased risk of T1D; more severe disease progression; more frequent severe diabetic ketoacidosis and weight loss in children with affected father	[100,101,102]
C-section delivery	Increased risk of T1D; reduced taxonomic and functional diversity of gut microbiome	[103]
Complicated vaginal delivery	Increased risk of T1D	[104]
Breastfeeding while introduction of the new food	Decreased risk of T1D	[104]
Short breastfeeding time	Increased risk of T1D	[105,106]
Early introduction of gluten-containing solid foods; introduction of solid foods early in life	Increased risk of islet autoimmunity	[104,107,108,109,110]
Fluctuations in the gut microbiome; high *Bacteroidetes*/*Firmicutes* ratio; decreased microflora diversity	Increased risk of T1D; rapid development of islet cell autoantibodies	[111,112,113,114,115,116,117,118,119]
Increased vitamin D consumption and higher serum 25-hydroxyvitamin D levels	Decreased risk of T1D	[120,121,122,123]
Coxsackie B virus, enteroviruses, and rotaviruses	Increased risk of T1D; rapid progression to T1D; activation of the immune system; destruction of pancreatic β-cells	[124,125,126,127,128,129,130]
Measles, and influenza vaccinations	Decreased risk of T1D	[131,132]
SARS-CoV-2 infection	Destruction of pancreatic β-cells	[133]
Early exposure to cow’s milk, and high consumption of cow’s milk	Increased risk of T1D; rapid progression to T1D	[134,135,136,137]
Higher consumption of sugars and carbohydrates	Increased risk of T1D; rapid progression to T1D	[138]
Higher birth weight and weight gain during the first year of life	Increased risk of T1D	[139,140,141,142]
Overweight, and obesity	Increased risk of T1D; association with high-risk HLA genotypes; increased risk of progression from islet autoimmunity to type 1 diabetes and with development of type 1 diabetes, but not with development of autoantibodies	[143,144,145,146,147]

## Abbreviations

ABIS	All Babies in Southeast Sweden;
ADA	American Diabetes Association;
BACH2	BTB Domain and CNC Homolog 2;
COVID-19	Coronavirus disease 2019;
DAISY	Diabetes Autoimmunity Study in the Young;
DiMe	Childhood Diabetes in Finland;
DiPiS	Diabetes Prediction in Skåne;
DIPP	The Finnish Type 1 Diabetes Prediction and Prevention;
FOXP3	Transcription factor forkhead box P3;
GADA	Autoantibodies to glutamic acid decarboxylase;
GRS	Genetic risk score;
HLA	Human leukocyte antigen;
HR	Hazard ratio;
IA-2A	Autoantibodies to islet antigen-2;
IAA	Autoantibodies to insulin;
ICA	Islet cells antibody;
INS	Insulin gene;
VNTR	Variable number of tandem repeats;
INSPAD	International Society for Pediatric and Adolescent Diabetes;
OGTT	Oral glucose tolerance test;
OR	Odds ratio;
PTPN22	Protein tyrosine phosphatase, non-receptor type 22 gene;
SARS-CoV-2	Severe acute respiratory syndrome coronavirus 2;
SLC30A8	Solute Carrier Family 30 Member 8;
SNP	Single nucleotide polymorphism;
TEDDY	The Environmental Determinants of Diabetes in the Young;
T1D	Type 1 diabetes;
Treg	Regulatory T Cell;
ZnT8A	Autoantibodies to zinc transporter 8
